# CH/π-interaction-driven self-assembly of tetraphenylethylene derivatives into the face to face arrangement†

**DOI:** 10.1039/d0ra10572d

**Published:** 2021-01-12

**Authors:** Lirong Yu, Mengxing Zhang, Dandan Lou, Jiale Li, Xi Wang, Ming Bai

**Affiliations:** Marine College, Shandong University, Weihai Weihai 264209 People's Republic of China xi_wang@sdu.edu.cn ming_bai@sdu.edu.cn; SDU-ANU Joint Science College, Shandong University, Weihai Weihai 264209 People's Republic of China

## Abstract

For tetraphenylethene (TPE) derivatives, it is difficult to determine the arrangement of the molecules in the aggregation state because disordered aggregation usually occurs. To solve the problem, we have explored a novel and facile strategy to investigate the aggregation mode of a TPE derivative framework in which the two neighboring *ortho* carbons of two phenyl moieties at the same ethylene carbon were linked with an alkoxyl chain (C4) (denoted as TPEC4). The XRD measurements on the particles obtained in a DMSO/H_2_O mixture (*f*_w_ = 60%) showed sharp peaks which is consistent with the simulated XRD patterns on the basis of a single crystal structure of TPEC4, indicating well-ordered molecular packing in the aggregated state. The CH/π-interaction and solvophobicity driven self-assembly behaviour of the compound was observed in the DMSO/H_2_O mixture. A face to face molecular packing structure that arises from quadruple intermolecular CH/π-interactions of the tetraphenylethylenes is the key motif for self-assembly in solution. The unique blue-red shifted emission in the DMSO/H_2_O mixture associated with aggregated behaviour of the compound was also investigated. This discovery will provide the basis for theoretical research and the rational design of TPE-based luminogens.

## Abstract 2

The CH/π interaction can drive the TPE motif to adopt a face-to-face crystalline packing in this TPE benzene rings linked with an alkoxyl chain (C4) during aggregation.

## Graphical abstract

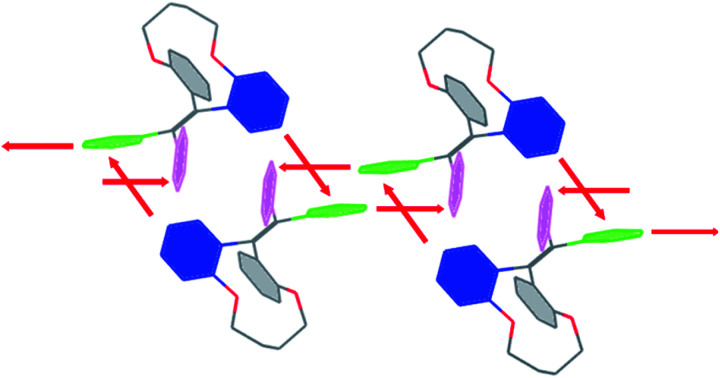

## Introduction

Tetraphenylethene (TPE) derivatives have been attracting increasing research interest in the past decade because of their intriguing aggregation-induced emission (AIE) properties.^1–6^ The intramolecular rotation, vibration and photoisomerization *via* a π bond twist of TPE luminogens have been efficiently suppressed in the solid state, which then blocks the radiationless relaxation channel, opens the radiative decay pathway,^7,8^ and allows TPE to emit efficiently in a concentrated state, endowing TPE derivatives with diverse application potentials in organic light-emitting, biosensing, fluorescent sensors, and biological probes.^9^

As aggregation induced emission luminogens, the driving forces or the methodologies of aggregation are very important not only because they can open the radiative decay pathway but also because the packing status in the aggregate state could affect the emission properties of AIE luminogens.^10,11^ To aggregation process, the AIE luminogens, especially TPEs, were typically driven self-organized singly or multiply by weak non-covalent interactions, such as solvophobic effects, host–guest interaction, metal–ligand coordination, intermolecular π–π interactions, electrostatic interactions, and hydrogen bonding interactions *etc.*^12,13^

CH/π interactions, a type of weak hydrogen bond, in which the energy associated with CH/π interactions are much smaller (0.8 kcal mol^−1^) relative to that of typical strong hydrogen bonding interactions between H–O⋯H or H–N⋯H (3.5 to 7.8 kcal mol^−1^) and the average distance between the C-donor and π-acceptor was observed to be longer than 2.5 Å.^14^ But these weak bonds contribute significantly to the conformation and stability of 3D structures of biological macromolecules as well as in many molecular recognition events.^15,16^ Interestingly, it was also found to be strong enough to drive molecules in a particular conformation to produce higher-order self-assembly.^14,17–19^ Although massive intermolecular CH/π interactions exist which can help to restrict the free rotational motions of the phenyl rings of TPE,^10,11^ the CH/π-interaction-induced molecular self-assembly in the TPE systems has not been reported.

In our previous work, the alkoxyl chains with different length ranging from one to four carbons are employed to link the two phenyl moieties on one end of ethylene in the TPE frameworks, resulting in a successful regulation over the molecular conformation.^20^ Among the series compounds, the compound TPEC4 showed a unique blue-red shifted emission property in mixtures of H_2_O/DMSO with different water fractions (*f*_w_, the volume percentage of H_2_O in the H_2_O/DMSO mixture). In this work, combing the powder XRD patterns of the particles formed in DMSO/H_2_O mixture with *f*_w_ = 60% and the single crystals structure of TPEC4 which was obtained in mixed THF and methanol (3/1, v/v), we found that in the aggregation state with shorter wavelength emission, the two TPEC4 enantiomers adopt a face to face arrangement with a lightly offset in crystal state. Encouragingly, the quadruple CH/π interactions exist between the adjacent TPE molecules which dominate the self-assembly of the TPEC4 molecules together with solvophobic effect. The relation between the luminescent property and the structure was further investigated.

## Experimental

### Reagents

Tetrahydrofuran (THF) was distilled from sodium wire and benzophenone under nitrogen. Dichloromethane (DCM) was distilled from CaH_2_. Column chromatography was carried out on a silica gel column (Qingdao Haiyang, 200–300 mesh) with the indicated eluents. All the other reagents such as benzophenone, *n*-butyllithium in hexane (2.5 mol L^−1^), 2,2′-dihydroxybenzophenone, diphenylmethane, methyl iodine, diiodomethane, 1,2-dibromoethane, 1,3-dibromopropane, 1,4-dibromobutane were used as received.

### Instrumentation

^1^H NMR spectra were recorded on a Bruker DPX 400 spectrometer (^1^H: 400 MHz, ^13^C: 100 MHz) in CDCl_3_. Spectra were referenced internally using the residual solvent resonances (*δ* = 7.28 for ^1^H NMR) relative to SiMe_4_ (*δ* = 0 ppm). ^13^C NMR spectra were referenced internally by using the solvent resonances (*δ* = 77.00 ppm for CDCl_3_). Electronic absorption spectra were recorded on a Hitachi U-2900 spectrophotometer. Steady-state fluorescence spectra were recorded on a Hitachi F-7000 spectrophotometer and an Edinburgh Instruments FLS920 three-monochromator spectrophotometer. The emission spectra were corrected for the wavelength dependence of the sensitivity of the detection system. The absolute fluorescence quantum yields were measured with an integrating sphere. Fluorescence images were taken on a Nikon Eclipse Ti florescence microscope. ESI-MS spectrum was taken on a Thermo Fisher Q-Exactive mass spectrometer. Single-crystal X-ray diffraction analyses were performed on an Aglient Super Nova Atlas Dual diffractometer using CuKα radiation (*λ* = 1.54184 Å). Structure was solved by direct methods using SHELXTL and refined by full-matrix least-squares on *F*^2^ using SHELX-97. Non-hydrogen atoms were refined with anisotropic displacement parameters during the final cycles. Hydrogen atoms were placed in calculated positions with isotropic displacement parameters set to 1.2 × *U*_eq_ of the attached atom. Crystallographic data (excluding structure factors) for the structures reported in this paper have been deposited in the Cambridge Crystallographic Data Center with CCDC Number: 2019817.

### ^1^H NMR and ESI-MS

Compound TPEC4. Yield: 92% (white powder). ^1^H NMR (400 MHz, CDCl_3_), *δ* (TMS, ppm): 7.08–7.04 (m, 14H, ArH), 6.79 (t, 2H, *J* = 7.2 Hz, ArH), 6.75 (d, 2H, *J* = 8.0 Hz, ArH), 4.33 (s, 2H, –OCH_2_–), 4.11 (s, 2H, –OCH_2_–), 1.97 (s, 2H, –CH_2_–), 1.97 (s, 2H, –CH_2_–). ^13^C NMR (100 MHz, CDCl_3_), *δ*: 156.03, 142.97, 142.17, 134.52, 134.03, 131.09, 130.48, 127.58, 127.10, 126.04, 120.71, 115.31, 69.96, 26.97. ESI-MS: found an isotopic cluster peaking at *m*/*z* [M + H^+^] 419.17; calculated for C_30_H_26_O_2_, 419.20.

## Results and discussion

### Luminescent properties of TPEC4 in DMSO/H_2_O mixture

**Fig. 1:**
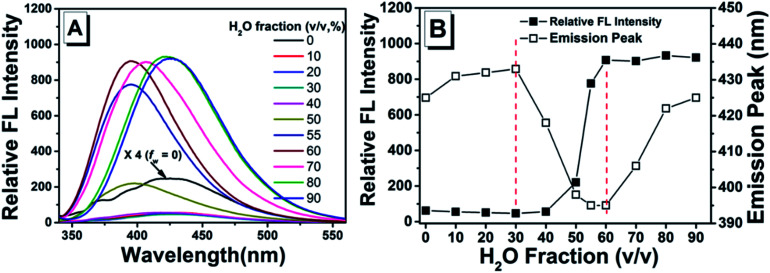
(A) Fluorescence spectra and (B) plots of the emission peak and fluorescence intensity at maximum emission of TPEC4 in DMSO/H_2_O mixtures with different water fractions. Concentration: 50 μM; *λ*_ex_: 330 nm (5 nm, 5 nm); 293 K.

As shown in previous work, to investigate the AIE activity and the emission properties of TPEC4, the fluorescence and electronic absorption spectra in DMSO/H_2_O with different water fractions were studied.^20^ As shown in Fig. 1, a weak emission at 425 nm could be found in the enlarged spectrum of TPEC4 in DMSO due to the restriction in part for the alkoxyl chain. When the *f*_w_ increases from 0 to 30%, the emission showed a slightly red shifted emission from 425 to 433 nm without obvious changes in intensity. With the *f*_w_ increasing from 30% to 60%, the fluorescence emission gets increased in a swift manner. A blue-shifted emission from 433 to 395 nm was observed. But the value of *f*_w_ from 0 to 55%, the absorption band of the solution does not change obviously with *λ*_max_ ≈ 286 nm (Fig. S1, ESI†) which is not consisted with the phenomena of typical H-aggregation. After the *f*_w_ gets over 70%, the TPEC4 exhibits a red-shift emission from 395 to 425 nm again with the absorption *λ*_max_ changed from 286 to 292 nm. The wavelength of emission peak in *f*_w_ = 90% is identical to *f*_w_ = 0. The fluorescence spectra of TPEC4 in DMSO in different concentration (0.5 to 800 μM) were also recorded (Fig. S2, ESI†). With the concentration increasing, the emission peak located at about 425 nm with increasing intensity. By plotting the fluorescence intensity *versus* the concentration, a nonlinear line is obtained (Fig. S2B, ESI†), indicating that compound TPEC4 is aggregated under these conditions.

### Structure and luminescent properties of TPEC4-THF compared with TPEC4-DCM in crystal state

**Fig. 2:**
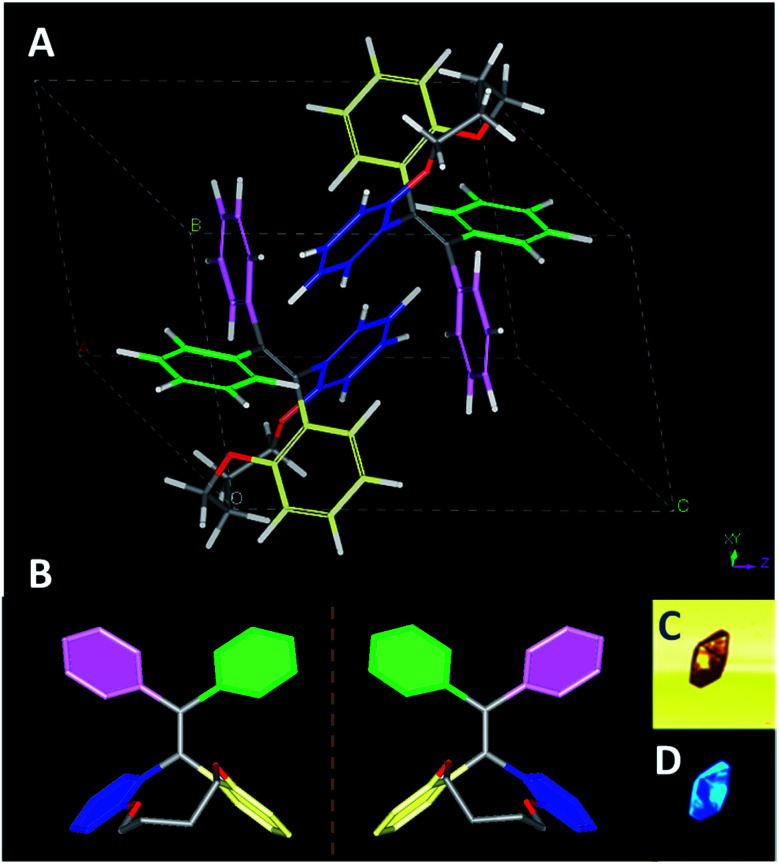
(A) The structure of TPEC4-THF molecular in a unit cell and (B) a pair of conformational enantiomer existed in a unit cell with non-related hydrogens omitted for clarity; the images of crystals under bright field (C) and UV-light irradiation (D) (wavelength: 330–380 nm).

Fortunately, the single crystals of TPEC4 were obtained in mixed THF and methanol (3/1, v/v) *via* slow evaporation (denoted as TPEC4-THF). The packing model of the TPEC4 molecules in crystal is different from our previous report, in which the reported crystal is obtained in CH_2_Cl_2_ and methanol mixed solvent (TPEC4-DCM; CCDC number: 1820144; Fig. S7, ESI†).^20^ The detailed crystallographic data of the two kinds of crystals which crystallized in different space groups are listed in Table S1 (ESI†). As shown in Fig. 2A and B, compound TPEC4-THF exists as a pair of conformational enantiomers in a unit cell. Table S2 (ESI†) compares the dihedral angles formed between the four phenyl moieties and the C=C bond of TPEC4 in *P* conformation (Fig. S5†) (the nomination of the conformational enantiomers is presented in Fig. S4, ESI†). The dihedral angles between the four phenyl rings and the C=C bond in TPEC4, ranging from 38.071(106) to 68.978(72)° (Table S2, ESI†). No obvious luminescence of the single solid crystals was observed under bright field, however, the single crystals become to emit strong blue light under UV irradiation (wavelength: 330–380 nm) (Fig. 2C and D).

**Fig. 3:**
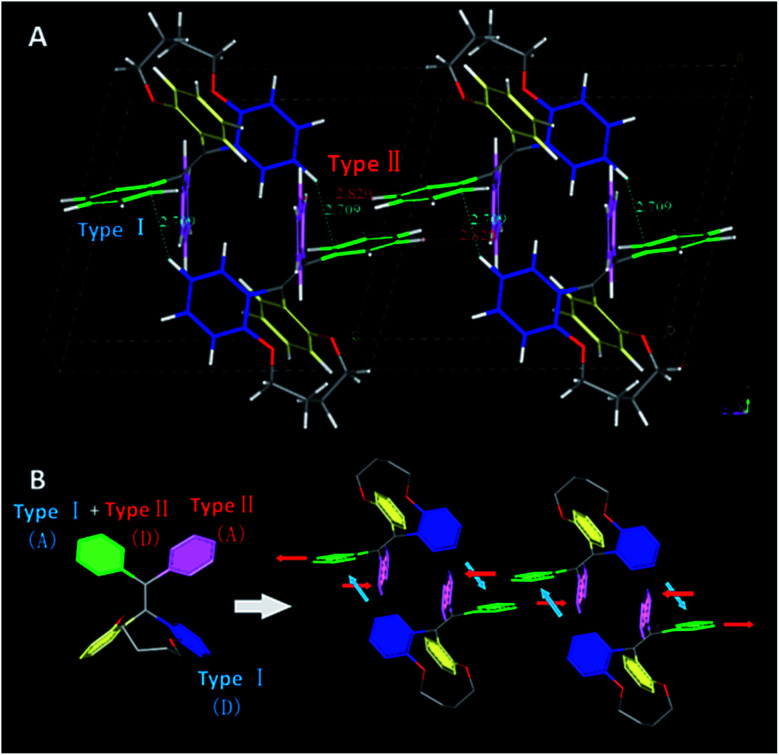
The intermolecular CH/π-interactions in TPEC4-THF (A), including the interaction within one unit cell (Type I) and the interaction between the adjacent unit cells (Type II); schematic representation of the CH/π-interactions that leads to the self-assembly of TPEC4-THF (B).

**Fig. 4:**
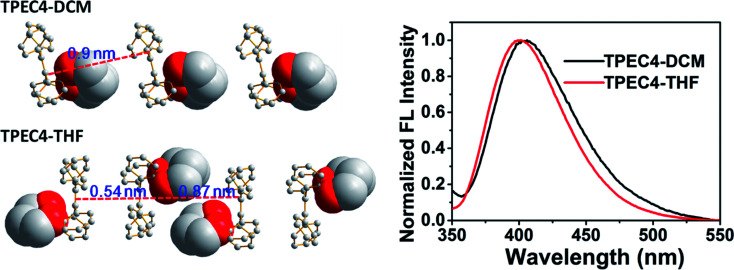
The packing modes of TPEC4 in TPEC4-DCM and TPEC4-THF with non-related hydrogens omitted for clarity and normalized fluorescence spectra of TPEC4-DCM and TPEC4-THF in crystal state. *λ*_ex_: 330 nm (5 nm, 5 nm); 293 K.

To point out the driven force for such arrangement of TPE moiety in TPEC4-THF, the intramolecular/intermolecular interaction was further investigated. The intramolecular hydrogen bonding interactions of C–H⋯O (2.725 Å) existed in each molecule, however, no typical intermolecular hydrogen bonds were observed (Fig. S6, ESI†). Therefore, the other weak non-covalent interactions could possibly induce the assembly of the compound with such twist molecular conformation. It can be seen from Fig. 3A, there are two different types of CH/π interactions existed in this compound, including the interactions within one unit cell and between adjacent unit cells. In one unit cell, CH/π interactions occur in a nearly vertical direction between the hydrogen atoms (2-position) of one benzene ring linked to the alkoxyl chain (donor) and the benzene plane arranged at the diagonal position of the former benzene ring in the adjacent molecule (acceptor). The resulted nonbonding H⋯C distances is about 2.709 Å and such interaction is donated as Type I CH/π interaction. Between the adjacent unit cells, the C–H bond located at 2-position of the benzene ring which act as an acceptor in the above Type I interaction is nearly perpendicular to the neighbouring benzene plane. Between the neighbouring molecules, the shortest nonbonded distance between H provided by C–H bond (2-position) and C existed in the vertical plane is 2.820 Å. This C–H-donor/π-acceptor system is the second kind of CH/π-interactions, which donated as Type II interaction. Fig. 3B shows schematic representation of the CH/π-interactions clearly, in which the staggered quadruple intermolecular CH/π-interactions formed. That is, the two kinds of different perpendicular t-shaped CH/π bonds between the adjacent molecules distributed throughout the whole structure. To the best of our knowledge, generally, TPE molecules were regarded as adopting highly twisted molecular conformations which reduced or eliminated the face to face π–π stacking between the adjacent TPE molecules.^21^ In this structure, similarly, the dominated CH/π interaction make the two molecules of TPEC4-THF adopted a face to face arrangement with a lightly offset. It is worth mentioning further that besides the weak non-covalent interactions play an important role in the self-assembly, the solvophobic effect should also be considered. The distinctly different packing model of the TPEC4-THF and TPEC4-DCM crystal can be used as evidence for this point. Different from the CH/π interaction in TPEC4-THF, only intermolecular/intramolecular hydrogen bonding interactions existed in TPEC4-DCM (Fig. S6 and S7, ESI†). Compared with the face to face arrangement of TPE moiety in TPEC4-THF, the different one by one form was observed in the structure of TPEC4-DCM. To the compound TPEC4, the alkoxyl linkage was connected on the two neighboring *ortho* carbons of the two phenyl moieties at the same ethylene carbon which could form a steric hindrance on one side of the TPE plane. When the TPEC4 molecules packing, the one side bulky group can separate the two adjacent TPE cores, as the result, the TPEC4 molecules will arrange one by one or face to face which consist with the two crystal structures of TPEC4 (Fig. 4). The distance of the two C=C bonds of TPEC4 molecules are *ca*. 0.54 nm (TPEC4-THF) and *ca.* 0.90 nm (TPEC4-DCM) separately. Generally, the luminescent property is closely related to the package of TPE moiety. However, no obviously different fluorescence spectra of TPEC4-DCM (*λ*_em_ = 405 nm) and TPEC4-THF (*λ*_em_ = 400 nm) in crystal state were observed (Fig. 4). The blue-shifted emissions of two kind of crystals mainly attribute to the similar geometrical rigid crystal environment which could be proved by the following evidences. (1) Comparing the dihedral angles between the four phenyl rings and the C=C bond, the conformations of the TPEC4 in TPEC4-DCM and TPEC4-THF are similar which consists their emission properties. The packing models of the TPEC4 molecules adopted have limited effects on their emission. (2) The absorption spectra did not change obviously when the emission shifted to shorter wavelength. In the UV-Vis spectrum, electrons are absorbing energy means that the electrons are going to excited state from its ground state, which indicates that the TPEC4 is having energy band gap. Therefore, the energy band gap can be determine by absorption wavelength. As shown in the UV-Vis spectrum (Fig. S1, ESI†), the spectra are almost the same, except the abnormal absorption spectra (*f*_w_ = 55, 60%) which may attribute to the particles formed in these situations make the scattering greater than the absorption. Thus, the fact of the similarity in the absorption indicates that the energy gap of the TPEC4 was not affected by the assembly.

### Luminescent properties and aggregation behaviour of the TPEC4 in DMSO/H_2_O mixture

**Fig. 5:**
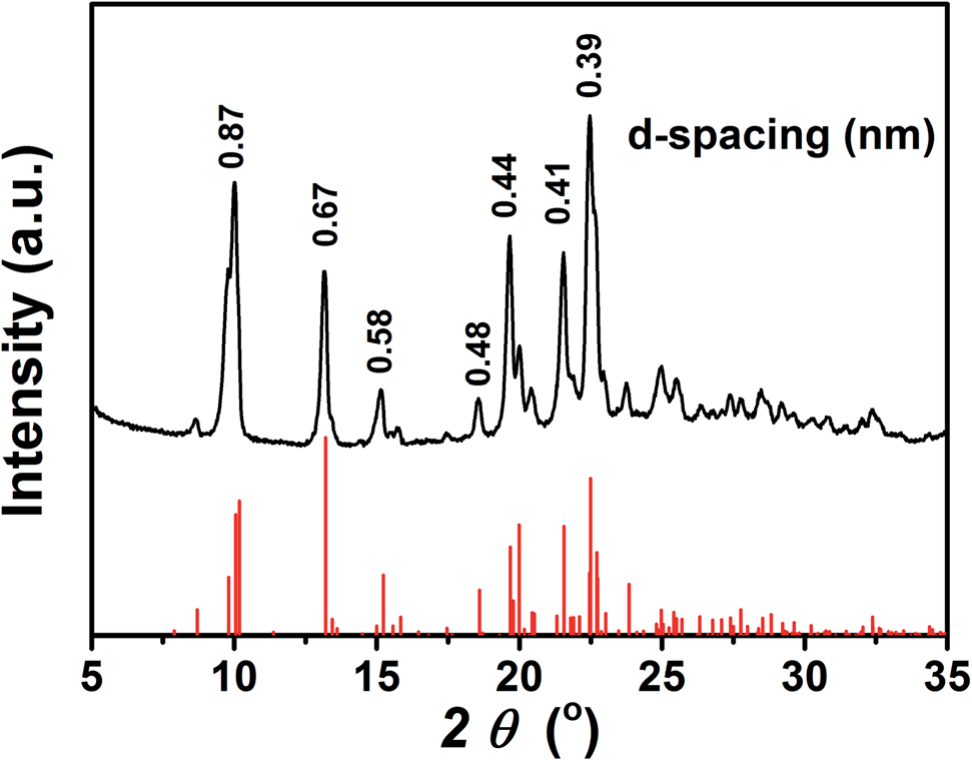
XRD pattern of TPEC4 particles formed from DMSO/H_2_O mixture (*f*_w_ = 60%) (black), together with the simulated powder diffraction pattern on the basis of X-ray crystallographic data (red).

To further investigate the aggregation behaviour of the TPEC4 with blue-shifted emission, the particles formed in DMSO/H_2_O mixture with *f*_w_ = 60% (concentration: 50 μM) were obtained by centrifugation. Fig. 5 shows that the XRD patterns of the as prepared freeze-dried sample is identical with the simulated powder diffraction pattern on the basis of X-ray crystallographic data of the TPEC4-THF crystals, which indicated that the molecular packing of TPEC4 with blue-shifted emission is an ordered face to face structure. To determine the structure of the aggregated particles with red-shifted emission under higher water fraction, the XRD pattern of the particles formed in DMSO/H_2_O mixture with higher *f*_w_ (90%) was obtained. Several sharp peaks appeared in the XRD pattern, implying that the aggregates (*f*_w_ = 90%) had a certain degree of crystallinity (Fig. S8, ESI†). It was also found that the peaks of XRD patterns were assigned to both the simulated XRD pattern of TPEC4-DCM and TPEC4-THF crystal. As mentioned above, the CH/π interaction and intermolecular/intramolecular hydrogen bonding interactions existed in TPEC4-THF and TPEC4-DCM crystal respectively. Thus, the mixed XRD pattern indicates that the self-assembly in the solution (*f*_w_ = 90%) was induced by multiple interactions, in which the CH/π and intermolecular/intramolecular hydrogen bonding interactions. As a result, various aggregation packing modes induced the mixed broad emission spectrum. In addition, the disordered aggregation also occurred in this condition (*f*_w_ = 90%), leading to that the excited and ground states was not strictly limited by the crystal environment anymore. Therefore, the Stokes shift increased and the red shift in the emission band is observed.

**Fig. 6:**
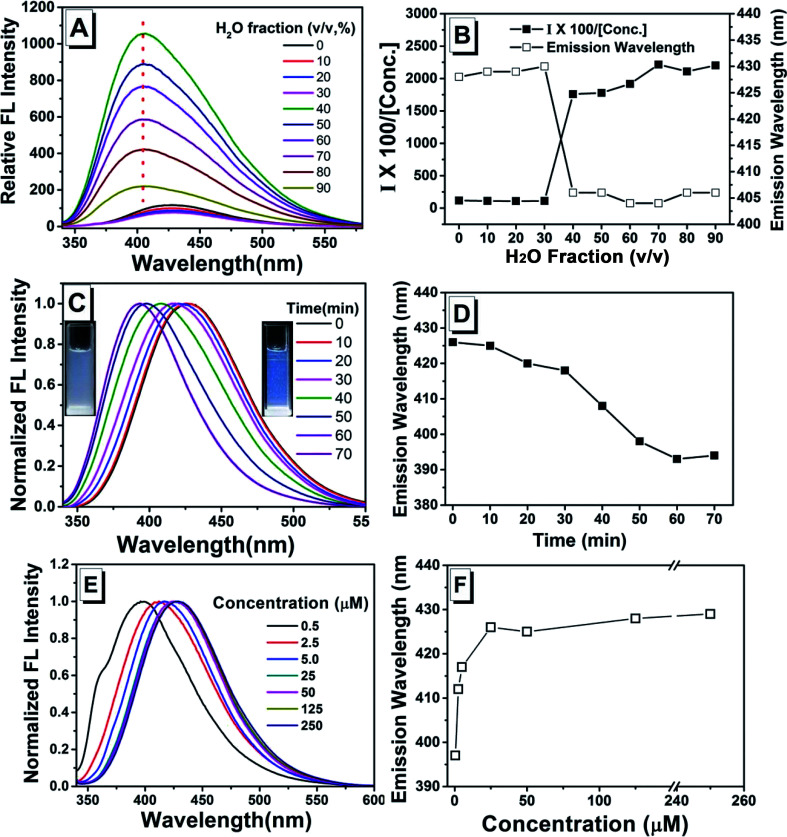
(A) Fluorescence spectra and (B) plots of the emission peak and fluorescence intensity at maximum emission of TPEC4 in DMSO/H_2_O mixtures with different water fractions in which the water was added continuously (concentration: from 100 to 10 μM); (C) the normalized spontaneous change of fluorescence spectra of TPEC4 in DMSO/H_2_O mixture (*f*_w_ = 90%) (concentration: 50 μM) and (D) the plots of the emission wavelength against time; (E) the fluorescence spectra of TPEC4 in DMSO/H_2_O mixture (*f*_w_ = 90%) in different concentration (from 0.5 to 250 μM) and (F) the plots of the emission wavelength against concentration. *λ*_ex_: 330 nm (5 nm, 5 nm); 293 K.

To clarify the role of assembly process, the fluorescence spectra of TPEC4 with the H_2_O was consecutively added into the stock solution in DMSO (100 μM) were recorded (the volume of H_2_O added each time and the *f*_w_ were listed in Table S3†). The emission intensity does not show obvious changes when the *f*_w_ increases from 0 to 30% (Fig. 6A and B). With the *f*_w_ increased to 40%, TPEC4 exhibits a blue-shifted emission from *ca.* 425 to 405 nm. But the blue-shifted emission peaks are broad which indicate that the emissions are multiple components. The absorption of the TPEC4 does not change with *λ*_max_ ≈ 286 nm when the *f*_w_ increases from 0 to 40% which consists with the result mentioned above. But the emission peak does not exhibit red-shift with the *f*_w_ increasing further which could contribute to the amorphous aggregation process was depressed with the H_2_O consecutively added. Consisting with this conclusion, the emission of TPEC4 in H_2_O–DMSO mixture (*f*_w_ = 90%) was found to be time dependent. As shown in Fig. 6C and D, the emission shows a spontaneous blue-shift change from 425 to 393 nm and the solution change from suspension to emulsion. This is a typical derived assembly process that the TPEC4 molecules in amorphous state were solvated and assembled in a face to face packing driven by the CH/π interactions together with the solvophobic effects. Correspondingly, the fluorescence spectra of fresh prepared TPEC4 in DMSO–H_2_O mixtures with *f*_w_ = 90% are concentration dependent. As shown in Fig. 6E and F, with the concentration increasing from 0.5 to 250 μM, the emission peak shifted from 397 to 429 nm. The amorphous aggregation at low concentration will be depressed and predominated at high concentration.

## Conclusions

In summary, for AIE luminogens, the aggregation packing mode and the driving force have important effects on their emission. Here, in TPEC4, the quadruple CH/π interaction could drive the TPE molecules adopting a face-to-face crystalline packing. The short wavelength emission mainly attributes to the similar geometrical structure at excited and ground states which was limited by the rigid crystal environment. The blue-shifted phenomena in spectra is attribute to this kind of aggregation predominate during the aggregation process. It is difficult to obtain a uniform aggregation structure using the method that the solvent changing from good to poor. The mixed emission derived from various aggregation packing modes is the main reason for the inconsistent fluorescence emission peaks in this article.

## Conflicts of interest

There are no conflicts to declare.

## Supplementary Material

RA-011-D0RA10572D-s001

RA-011-D0RA10572D-s002
