# Supplementary material for: CH/π-interaction-driven self-assembly of tetraphenylethylene derivatives into the face to face arrangement
Source: RSC Adv. 2021 Jan 12;11(4):2377–82. doi: 10.1039/d0ra10572d (PMC8693644; doi:10.1039/d0ra10572d)
Supplement: RA-011-D0RA10572D-s001 [file RA-011-D0RA10572D-s001.pdf]

Electronic Supplementary Material (ESI) for RSC Advances.
This journal is © The Royal Society of Chemistry 2020

Supporting Information

CH/π-Interaction-Driven Self-Assembly of Tetraphenylethylene Derivatives into the Face to Face Arrangement

Lirong Yu, ‡[a] Mengxing Zhang, ‡[a] Dandan Lou, [a] Jiale Li, [a] Xi Wang* [a] and Ming Bai * [a,b]

[a] *Marine College, Shandong University, Weihai, Weihai 264209, People's Republic of China. E-mail: xi_wang@sdu.edu.cn; ming_bai@sdu.edu.cn;*

[b] *SDU-ANU Joint Science College, Shandong University, Weihai, Weihai 264209, People's Republic of China.*

‡ These authors contributed equally to this work.

Fig. S1. Uv-vis spectra of **TPEC4** in DMSO-H_2O mixtures with different water fractions. Concentration: 50 μM. The absorbance spectra of **TPEC4** at f_w = 55, 60% are not normal which may attribute to the particles formed in these situations make the scattering greater than the absorption.

Fig. S2. Fluorescence spectra of **TPEC4** in DMSO-H_2O mixtures with different water fractions in which the water was added continuously. (A) Fluorescence spectra and (B) plots of the fluorescence intensity at maximum emission of **TPEC4** in DMSO in different concentration (Concentration: from 0.5 to 800 μM).

Fig. S3. Uv-vis spectra of **TPEC4** in DMSO-H_2O mixtures with different water fractions in which the water was added continuously. Concentration: 100-10 μM. The absorbance spectra of **TPEC4** at f_w > 40% are not normal which may attribute to the particles formed in these situations make the scattering greater than the absorption.

Fig. S4. The rule for nomination of the two conformational enantiomers of **TPEC4**. The compounds possess propeller-shaped chirality (*P* and *M*) for achiral TPE twisting in one direction.

Fig. S5. The definition of the dihedral angles formed between the phenyl rings and the C=C bond.

Fig. S6. The intramolecular hydrogen bonding displayed in **TPEC4-THF** crystal. Blue and purple colored **TPEC4-THF** represent a pair of conformational enantiomers.

Fig. S7. The intramolecular/ intermolecular interaction (C-H···O hydrogen bonds) displayed in **TPEC4-DCM** crystal. Purple and yellow colored **TPEC4-DCM** represent a pair of conformational enantiomers.

Fig. S8. The XRD pattern of the particles obtained from f_w=90% (DMSO-H_2O) compared with the simulated XRD pattern of TPEC4-THF and TPEC4-DCM. The dot and asterisk assigned to the peaks in TPEC4-THF and TPEC4-DCM respectively.

Table S1. Crystallographic data of **TPEC4-THF** and **TPEC4-DCM**.

Table S2. The dihedral angles between the phenyl moieties and the C=C bond of **TPEC4-THF** and **TPEC4-DCM** with *P* conformation.

Table S3. The sample preparation for the **TPEC4** in DMSO-H_2O mixtures with different water fractions in which the water was added continuously (the stock solution is 100 μM, f_w =0%).

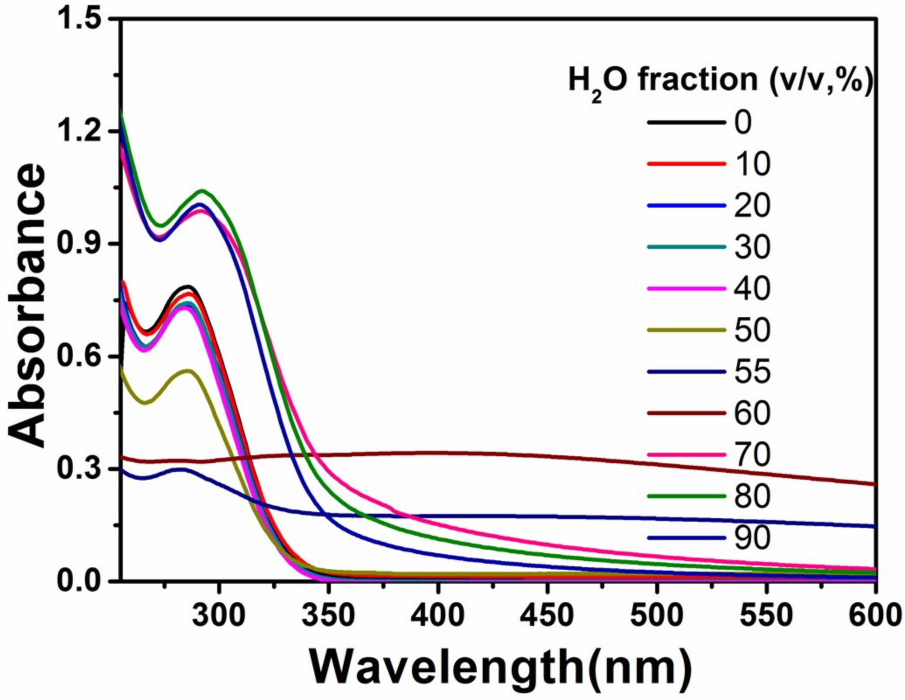

Fig. S1. Uv-vis spectra of **TPEC4** in DMSO-H_2O mixtures with different water fractions. Concentration: 50 μM. The absorbance spectra of **TPEC4** at f_w = 55, 60% are not normal which may attribute to the particles formed in these situations make the scattering greater than the absorption.

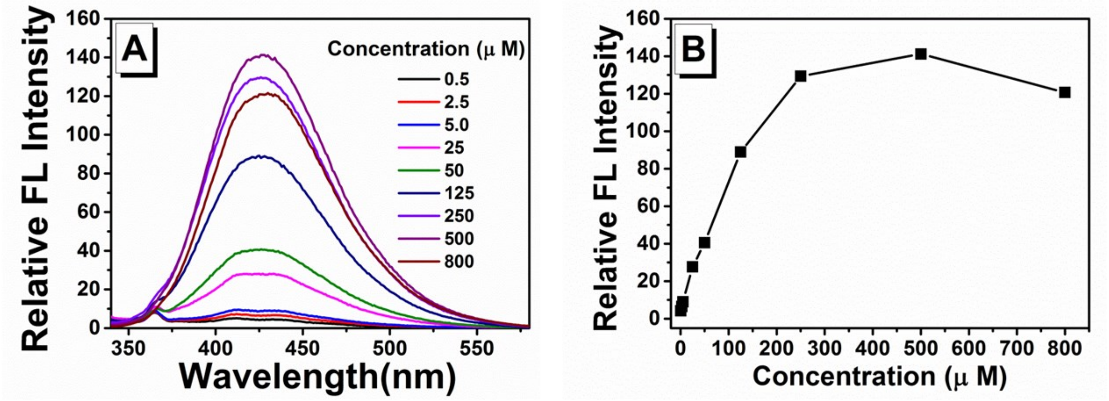

Fig. S2. Fluorescence spectra of **TPEC4** in DMSO-H_2O mixtures with different water fractions in which the water was added continuously. (A) Fluorescence spectra and (B) plots of the fluorescence intensity at maximum emission of **TPEC4** in DMSO in different concentration (Concentration: from 0.5 to 800 μM).

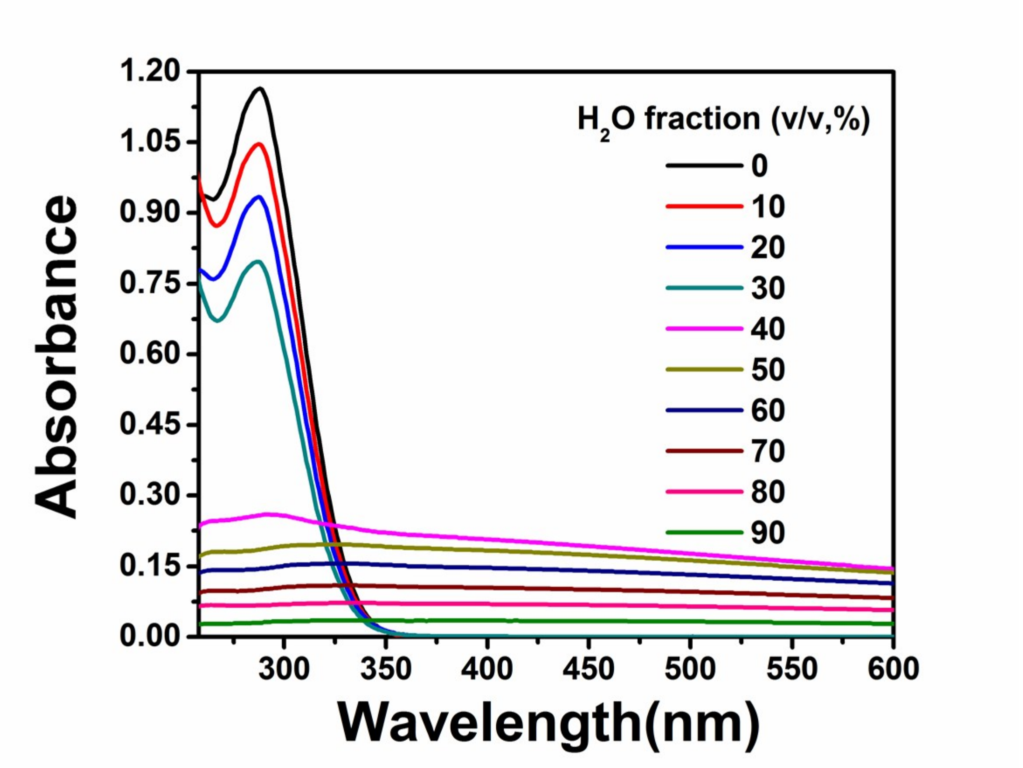

Fig. S3. Uv-vis spectra of **TPEC4** in DMSO-H_2O mixtures with different water fractions in which the water was added continuously. Concentration: 100-10 μM. The absorbance spectra of **TPEC4** at $f_w > 40\%$ are not normal which may attribute to the particles formed in these situations make the scattering greater than the absorption.

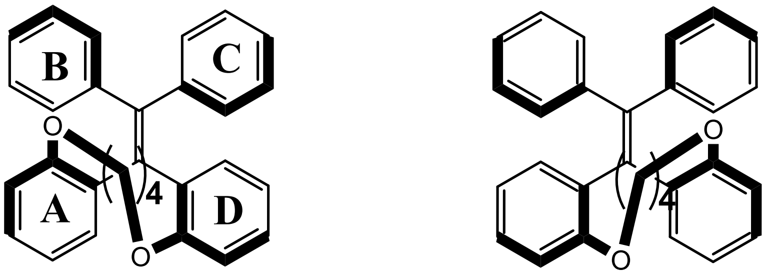

M-TPE **P-TPE**

Fig. S4. The rule for nomination of the two conformational enantiomers of **TPEC4**. The compounds possess propeller-shaped chirality (*P* and *M*) for achiral TPE twisting in one direction.

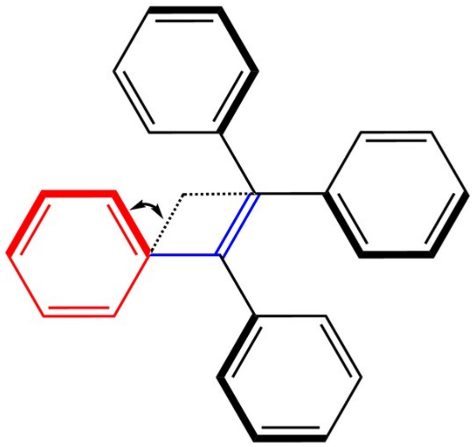

Fig. S5. The definition of the dihedral angles formed between the phenyl rings and the C=C bond.

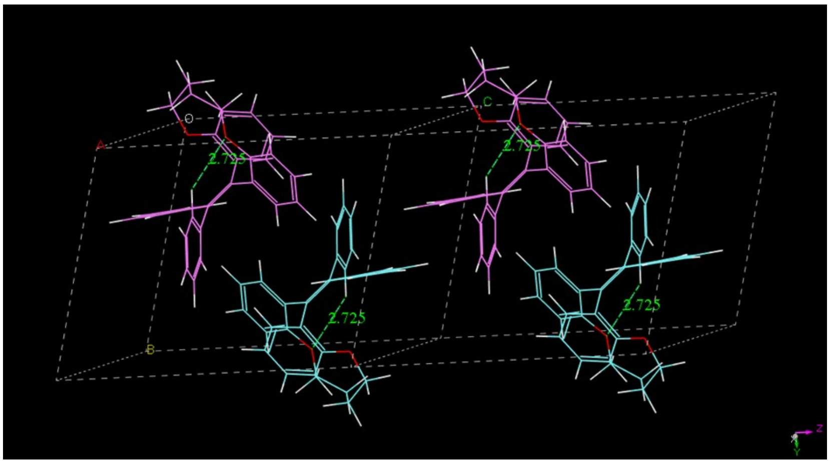

Fig. S6. The intramolecular hydrogen bonding displayed in **TPEC4-THF** crystal. Blue and purple colored **TPEC4-THF** represent a pair of conformational enantiomers.

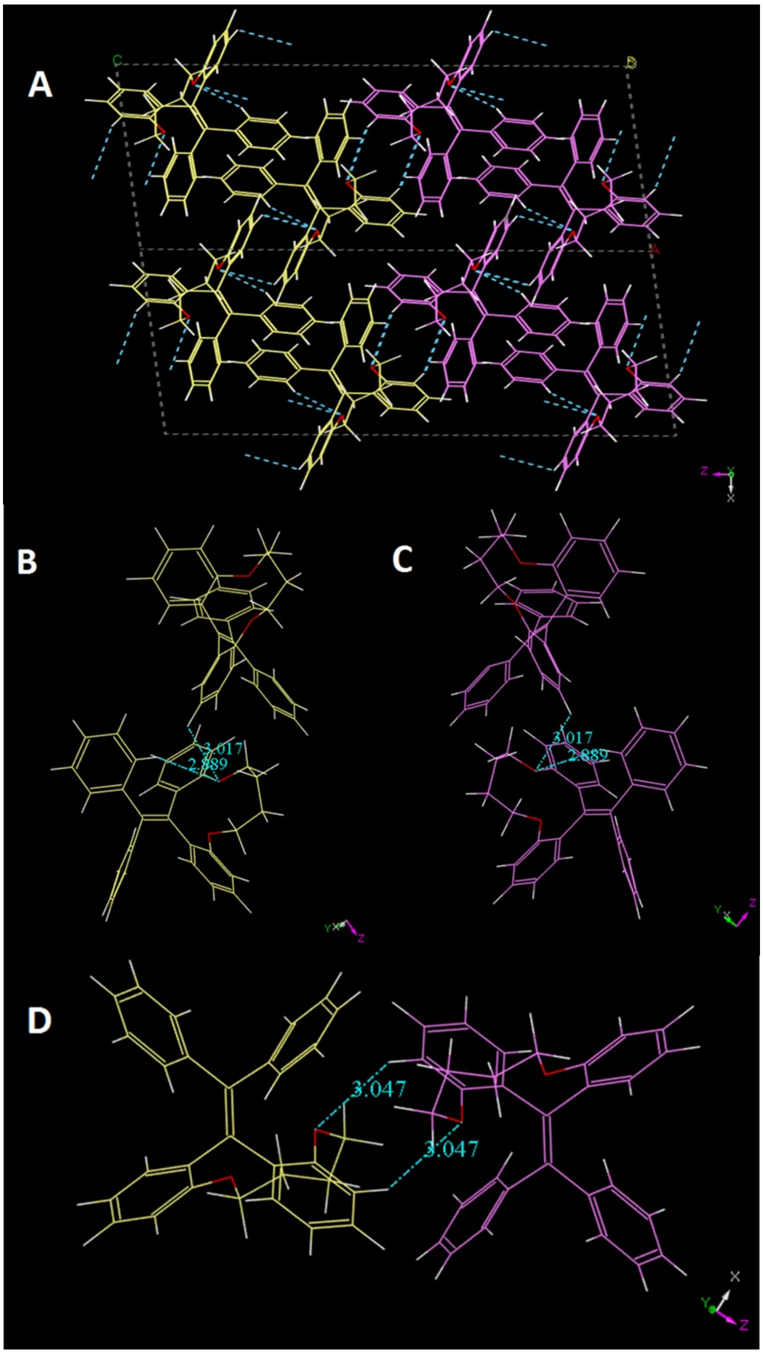

Fig. S7. The intramolecular/ intermolecular interaction (C-H···O hydrogen bonds) displayed in **TPEC4-DCM** crystal. Purple and yellow colored **TPEC4-DCM** represent a pair of conformational enantiomers.

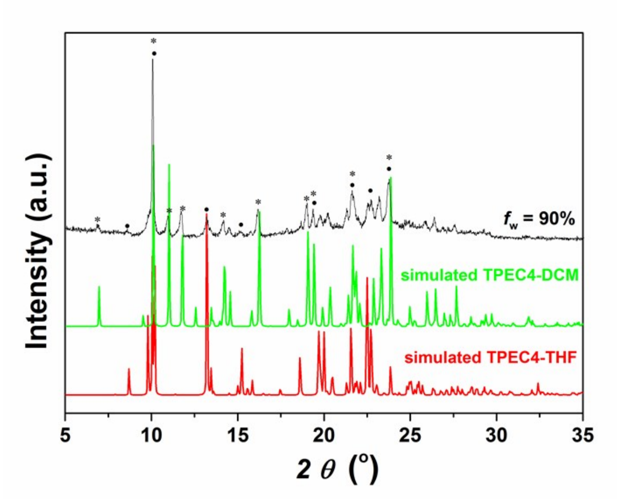

Fig. S8. The XRD pattern of the particles obtained from f_w=90% (DMSO-H_2O) compared with the simulated XRD pattern of TPEC4-THF and TPEC4-DCM. The dot and asterisk assigned to the peaks in TPEC4-THF and TPEC4-DCM respectively.

Table S1 Crystallographic data of **TPEC4-THF** and **TPEC4-DCM**.

Code	TPEC4-THF	TPEC4-DCM
Temperature(K)	293(2)	296(2)
Chemical formula	$C_{30}H_{26}O_2$	$C_{30}H_{26}O_2$
Crystal system	Triclinic	Monoclinic
Space group	P -1	$P2_1$/c
Formula weight	418.54	418.54
a(Å)	9.6474(7)	9.3824(12)
b(Å)	10.8318(6)	9.3181(12)
c(Å)	12.4029(9)	25.644(3)
α(°)	103.244(6)	90.00
β(°)	108.679(7)	97.845(4)
γ(°)	100.155(6)	90.00
Volume (Å^3)	1150.52(15)	2221.0(5)
D_c(gcm^{-3})	1.208	1.252
F(000)	444	888
Z	2	4
μ(mm^{-1})	0.577	0.077
R_1,[$I > 2\sigma(I)$]	0.0482	0.0731
R_1,(all data)	0.0575	0.1177
wR_2, [$I > 2\sigma(I)$]	0.1436	0.1638
wR_2, (all data)	0.1559	0.1806
GOF	1.046	1.030

Table S2. The dihedral angles between the phenyl moieties and the C=C bond of **TPEC4-THF** and **TPEC4-DCM** with *P* conformation.

Compound	A	B	C	D
**TPEC4-THF**	68.978(72)°	38.071(106)°	52.733(79)°	62.796(101)°
**TPEC4-DCM**	65.745(246)°	34.278(204)°	59.172(237)°	69.776(227)°

Table S3. The sample preparation for the **TPEC4** in DMSO-H_2O mixtures with different water fractions in which the water was added continuously (the stock solution is 100 µM, f_w =0%)

	**DMSO (mL)**	**Adding H_2O(mL)**	**H_2O (mL)**	**H_2O fraction (v/v)**	**Concentration (µM)**
1	5	0	0	0	100
2	5	0.556	0.556	10	90
3	5	0.694	1.25	20	80
4	5	0.893	2.143	30	70
5	5	1.19	3.333	40	60
6	5	1.667	5	50	50
7	5	2.5	7.5	60	40
8	5	4.167	11.667	70	30
9	5	8.333	20	80	20
10	5	25	45	90	10
